# The role of PET/CT in radiotherapy for nasopharyngeal carcinoma

**DOI:** 10.3389/fonc.2022.1017758

**Published:** 2022-10-21

**Authors:** Hongjia Li, Ziren Kong, Yongbo Xiang, Rong Zheng, Shaoyan Liu

**Affiliations:** ^1^ Department of Nuclear Medicine/PET-CT Center, National Cancer Center/National Clinical Research Center for Cancer/Cancer Hospital, Chinese Academy of Medical Sciences and Peking Union Medical College, Beijing, China; ^2^ Department of Head and Neck Surgery, National Cancer Center/National Clinical Research Center for Cancer/Cancer Hospital, Chinese Academy of Medical Sciences and Peking Union Medical College, Beijing, China; ^3^ Department of Radiation Oncology, National Cancer Center/National Clinical Research Center for Cancer/Cancer Hospital, Chinese Academy of Medical Sciences and Peking Union Medical College, Beijing, China

**Keywords:** PET/CT, nasopharyngeal carcinoma, radiotherapy, ^18^F-FDG, novel radiotracer

## Abstract

Radiotherapy has already been developed as the standard of care for patients with nasopharyngeal carcinoma (NPC), and precision staging, target volume delineation, prognosis prediction, and post-treatment surveillance are essential in the management of NPC. Positron emission tomography/computed tomography (PET/CT) is increasingly recognized as an imaging modality to guide precision radiotherapy in these areas. The feasibility and efficacy of ^18^F-FDG PET/CT have been confirmed in tumor diagnosis, treatment planning, prognosis, surveillance, and assessment. Coupled with the capability of revealing tumor metabolic information, ^18^F-FDG PET/CT is more accurate in identifying primary lesions and metastases of NPC than other conventional imaging methods including CT and MRI and shows the independently diagnostic and prognostic value for radiotherapy. However, ^18^F-FDG has limitations due to its physiological distribution in brain tissue and increasing uptake in post-radiation inflammation. Novel PET radiotracers including FAPI, NaF, CHO, and FLT are explored as alternatives with potential superiority for radiotherapy in NPC. In this review, we summarized the evolving role of PET/CT in the management of radiotherapy in NPC patients, aiming to facilitate precision radiotherapy from a molecular imaging aspect.

## Introduction

Nasopharyngeal carcinoma (NPC) is a special epithelial malignancy differing from other head and neck cancers. It arises from the nasopharyngeal mucosal lining and has three pathological subtypes: keratinizing squamous, non­keratinizing, and basaloid squamous. Non­keratinizing NPC can be further divided into differentiated and undifferentiated tumors. NPC is distinctly different from other epithelial head and neck cancers. The non­keratinizing subtype accounts for more than 95% of cases in endemic areas, whereas the keratinizing squamous subtype is relatively rare. The incidence rate of NPC is around 0.5-1.0/100,000 per year worldwide. It is prevalent in east and southeast Asia where the incidence rate is more than 3/100,000 per year (1, 2).

NPC has an insidious onset and is prone to early metastases. Different from other epithelial head and neck cancers, the anatomical structure of nasopharyngeal tumor lesions and surrounding tissues is complicated for complete surgical resection and brings challenges to treatment. Radiotherapy has been the mainstay of treatment modality and shows a significant curative effect for patients with NPC. It is exploited in all stages of tumors and significantly improves patient survival with a low recurrence rate.

CT, MRI, and PET/CT are prevailing imaging methods performed in patients with NPC for diagnosis, treatment planning, prognosis, surveillance, and assessment. Compared with CT and MRI, PET/CT has particular advantages as a new functional imaging modality and is increasingly applied in various conditions (3). Meanwhile, with the development of radiotracers, PET/CT is constantly improving its detection abilities and expanding clinical indications. It has become an irreplaceable imaging examination for patients with NPC. However, the definite superiority of PET/CT for guiding radiotherapy in NPC requires to be further confirmed and compared with conventional imaging methods including CT and MRI. In addition, various PET radiotracers that have been used for NPC are under investigation and show huge potential value. Therefore, this review aims to summarize the evolving role of PET/CT with different radiotracers in the management of radiotherapy in patients with NPC.

## PET radiotracers

Positron emission tomography/computed tomography (PET/CT) is a functional or molecular imaging procedure allowing for the three-dimensional mapping of positron emitting radiopharmaceuticals in contrast to computed tomography (4). Radionuclide-labeled tracers deliver excellent signals to target and achieve molecular imaging results. Consequently, with the development of nuclear medicine, PET/CT has a wide range of applications in tumors and is increasingly used in clinical procedures and research of NPC.

In most cases, ^18^F-fluorodeoxyglucose (^18^F-FDG) is utilized as the radiotracer for PET/CT in patients with NPC. As a glucose analog labeled by an isotope of Fluorine, ^18^F-FDG is transported into cells and phosphorylated by hexokinase without undergoing further metabolism. The uptake of ^18^F-FDG reflects the activity of cellular metabolism. Therefore, ^18^F-FDG could be used as an imaging marker for tumors with increased glucose uptake and glycolysis. Over the decades, ^18^F-FDG PET/CT has been successfully employed in tumor diagnosis, staging, treatment planning, prognosis, and assessment, but still has certain limitations due to its physiological distribution. ^18^F-FDG PET/CT is not able to observe skull-base involvement and intracranial extension, as well as brown fat throughout the head and neck region. Also, the detection rate for liver metastases is lower than MRI and contrast-enhanced CT. Novel PET radiotracers are urgently demanded to replace ^18^F-FDG in those clinical conditions.

Fibroblast activation protein (FAP) is a membrane-anchored peptidase and is highly expressed in the tumor microenvironment. FAP inhibitor (FAPI) coupled with chelator could specifically bind to fibroblast activation protein with rapid and complete internalization (5). Therefore, FAPI has high uptake in tumor tissue and low accumulation in normal tissue, providing a high contrast imaging for PET/CT. FAPI also has a rapid clearance from circulation which makes it operable and safe. The latest developments on FAPI structures used in clinical applications include FAPI-02, FAPI-04, FAPI-34, FAPI-46, and FAPI-74, labeled with ^68^Ga and ^99m^Tc for diagnosis as well as ^188^Re and ^90^Y for treatment. FAPI has low uptake in brain, liver, oral and pharyngeal mucosa background and shows excellent imaging contrast. Currently increasing research has been conducted to explore its abilities and advantages in various cancers including NPC.

Sodium fluoride (NaF) is a radiotracer synthesized for nuclear imaging in the last century and is currently used in PET/CT for skeletal system imaging. ^18^F-NaF is rapidly cleared out with osseous uptake after injection and forms fluorapatite crystals into hydroxyapatite crystals in the extracellular bone matrix (6). The uptake of tracer is increased at sites with a high rate of bone remodeling and negligible in soft tissues. Therefore, ^18^F-NaF PET/CT is capable to identify bony neoplastic lesions and skeleton metastases with physiological deposition of the crystalline matrix surrounding tumors. Besides bone diseases, ^18^F-NaF is also used as a noninvasive quantitative imaging modality in other fields including pharmacokinetics and cardiovascular hemodynamics.

Choline (CHO) is a precursor of phosphatidylcholine which is an important component of the lipid bilayer structure of cell membranes. It is transported into cells, phosphorylated by choline kinases, and integrated into phosphatidyl-choline. Tumors with increased proliferation will stimulate a series of biosynthetic processes of cell membranes and eventually lead to increased uptake of choline. Labeled with ^11^C or ^18^F, CHO PET has been used in multiple cancers including brain cancers, prostate cancers, esophageal cancers, and lung cancers. It shows low uptake in normal brain tissues and can be especially used for neoplastic tissues without a high rate of glucose metabolism (7). ^11^C-CHO is the first radiolabeled choline but has short half-time decay. ^18^F-Fluorocholine (^18^F-FCH) is the fluorinated kind of choline including ^18^F-Fluoromethylcholine (^18^F-FMC) and ^18^F-Fluoroethylcholine (^18^F-FECH) with a longer half-life and better diagnostic accuracy.

Fluorothymidine (FLT) is another fluorinated amino acid tracer showing similar characteristics to CHO. As a thymidine analog, FLT is phosphorylated by thymidine kinase within cells and used for protein synthesis. The uptake of FLT is correlated with tumor cell proliferation and shows no increase in normal brain tissues (8). ^18^F-FLT PET is currently used for brain tumor imaging along with ^18^F-diidrossiphenilalanine (^18^F-DOPA) and ^18^F-fluorethyltirosine (^18^F-FET).

In general, ^18^F-FDG is still the most commonly utilized radiotracer for PET/CT in the routine radiotherapy process of NPC. FAPI, NaF, CHO, and FLT are novel radiotracers that have been explored and studied in recent research. Current evidence about PET radiotracers beyond ^18^F-FDG in patients with NPC is relatively rare but could provide additional information and show huge potential values.

## PET/CT in the TNM staging of NPC

TNM staging is fundamental for tumor diagnosis and treatment planning, along with the guidance to identify radiotherapy indications in patients with NPC. The staging is classified according to the 8^th^ edition of the American Joint Committee on Cancer (AJCC) staging system. Currently, MRI and PET/CT have been routinely applied in clinical practice for tumor staging with better diagnostic value in primary tumor extension and lymph node metastases than CT. Recent studies have confirmed PET/CT shows higher sensitivity, specificity, accuracy, positive predictive value, and negative predictive value for TNM staging of NPC (9). In Matsuura’s (10) study, 20% of patients with NPC changed TNM classification and 13% of patients changed clinical stages by ^18^F-FDG PET/CT compared with conventional imaging methods. In another study, 36% of patients changed T stages and 23% of patients changed N stages by ^18^F-FDG PET/CT compared with MRI (11). Early and accurate staging is vital for improving therapeutic effect and patient survival. Role of 18F-FDG PET/CT in NPC are summarized in **Table 1**.

**Table 1:** Critical studies investigating ^18^F-FDG PET/CT in NPC.

Author (year)	Patient number	Clinical stage	Treatment	Aspect	Notes
Xiao et al. (11) (2021)	872	I-II	RT, CCRT, ICT+RT, ICT+CCRT	Staging	^18^F-FDG PET/CT was significantly more sensitive in detecting cervical lymph node metastases and less sensitive in detecting retropharyngeal lymph node metastases and locoregional invasion than MRI.
Yang et al. (12) (2022)	1093	III	RT, CCRT, ICT+CCRT	Staging	^18^F-FDG PET/CT showed higher sensitivity and accuracy in diagnosing lymph nodes than MRI. Patients that underwent both ^18^F-FDG PET/CT and MRI had better 5-year OS than those underwent MRI alone.
Wu et al. (13) (2016)	20	I-II	RT, CCRT	Radiotherapy planning	This study confirmed the feasibility of ^18^F-FDG PET/CT guided IMRT. GTVs showed no differentiation but PTVs were delineated more accurately by ^18^F-FDG PET/CT than by CT and MRI.
Matsuura et al. (10) (2016)	124	I-IV	RT, CCRT	Radiotherapy planning	^18^F-FDG PET/CT significantly changed the target delineation for IMRT. The recurrent rate of groups guided by ^18^F-FDG PET/CT was lower than groups guided by CT, but the overall survival showed no significant difference.
Liu et al. (14) (2017)	213	III-IV	CCRT, ICT+CCRT, CCRT+ACT	Radiotherapy planning	Patients with radiotherapy guided by ^18^F-FDG PET/CT had significantly better OS than those by CT and no significant differences in toxicities were observed.
Hung et al. (15) (2013)	371	I-IV	RT, CCRT, ICT+CCRT	Prognosis	This study confirmed that SUVmax derived by ^18^F-FDG PET/CT had prognostic value for NPC. Patients with SUVmax of primary tumor lesions ≥9.3 and SUVmax of lymph nodes ≥7.4 had significantly worse DMFS.
Fei et al. (16) (2019)	82	I-IV	RT, CCRT, ACT+CCRT	Prognosis	This study confirmed that SUVmax and MTV derived by ^18^F-FDG PET/CT had prognostic value for NPC. Patients with SUVmax ≥8.2 and MTV ≥4.0 had significantly worse 3-year PFS.
Yoon et al. (17) (2016)	97	III-IV	RT, CCRT, ICT+CCRT	Prognosis	This study confirmed that TLG derived by ^18^F-FDG PET/CT had prognostic value for NPC. Patients with TLG ≥322 had significantly worse 5-year PFS.
Ma et al. (18) (2021)	29	I-IV (LRNPC)	CIRT	Prognosis	This study reported that HI derived by ^18^F-FDG PET/CT had potential prognostic value for LRNPC. Patients with HI ≥0.81 had significantly worse LRFS and PFS, whereas SUVmax, MTV, and TLG showed limited prognostic values in this study.
Jeong et al. (19) (2019)	143	I-IV	RT, CCRT, ICT+CCRT	Surveillance and assessment	^18^F-FDG PET/CT within 6 months after completely radiotherapy had high negative-predictive values for predicting residual disease and recurrence. Patients with SUVmax of lymph nodes ≥2.5 after radiotherapy had significantly higher risks of recurrence.
Chan et al. (20) (2013)	165	III-IV	CCRT	Surveillance and assessment	This study confirmed that ^18^F-FDG PET/CT after CCRT had independent prognostic value for local advanced NPC. 3-month post-therapy PET findings and TLG of primary tumor ≥330 were significantly associated with survival.

NPC, nasopharyngeal carcinoma; LRNPC, local recurrent nasopharyngeal carcinoma; RT, radiotherapy; CCRT, concurrent chemoradiotherapy; ICT, induction chemotherapy; ACT, adjuvant chemotherapy; IMRT, intensity-modulated radiotherapy; CIRT, carbon ion radiotherapy; GTV, gross tumor volume; PTV, planning target volume; SUV, standardized uptake value; SUVmax, maximum standardized uptake value; MTV, metabolic tumor volume; TLG, total lesion glycolysis; HI, heterogeneity index; OS, overall survival; PFS, progression-free survival; DMFS, distant metastases-free survival; LRFS, local recurrence-free survival.

The regional lymph node metastases are frequent in NPC and the involvement rate is up to 70-90%. PET/CT is sensitive for assessing lymph nodes especially those CT and MRI fail to detect. The pattern of regional lymph node metastases directly determines clinical staging and significantly influences radiotherapy planning and outcomes. Ng et al. (21) reported that ^18^F-FDG PET/CT was significantly more accurate than conventional imaging methods including CT and MRI in detecting cervical lymph node metastases of NPC and it facilitated a more appropriate radiotherapy planning for patients. Recently Xiao et al. (11) conducted a study that included 872 patients with NPC of stage I-II, showing similar results that ^18^F-FDG PET/CT was superior to MRI for detecting cervical lymph node metastases with higher sensitivity (96.6% *vs.* 76.4%, *p* < 0.001). Nevertheless, Yang et al. (12) analyzed 460 biopsied cervical lymph nodes and 1093 patients with NPC. In this study ^18^F-FDG PET/CT performed better than MRI with higher sensitivity, accuracy, and area under the receiver operating characteristic curve (96.7% *vs.* 88.5%, *p* < 0.001; 88.0% *vs.* 81.1%, *p* < 0.001; 0.863 *vs.* 0.796, *p* < 0.05) in diagnosing lymph nodes. Undergoing both ^18^F-FDG PET/CT and MRI prolonged the survival rate of patients with NPC compared with undergoing MRI alone.

Cervical lymph node metastases have a high incidence rate in early-stage NPC and are important for staging and identifying radiotherapy indications. However, PET/CT is less sensitive than MRI in detecting retropharyngeal lymph node metastases. Ng et al. (21) found that ^18^F-FDG PET/CT was less sensitive than MRI in detecting retropharyngeal lymph node metastases and resulted in downstaging for patients with NPC. Xiao et al. (11) also mentioned that ^18^F-FDG PET/CT is less accurate for the assessment of locoregional invasion and retropharyngeal nodal metastases in NPC with lower sensitivity than MRI (72.2% *vs.* 91.1%, *p* = 0.004). The failure in detecting retropharyngeal lymph node metastases may be due to the poor FDG uptake and early invasion of lymph nodes in this region.

NPC tends to spread early to local sites and has a higher incidence rate of metastases than other head and neck cancers (1). Previous studies showed that distant metastases are as frequent as 38–87% in patients with NPC, and 11-36% of patients present overt distant metastases at initial diagnosis (22, 23). Early metastases detection and precise staging are essential for the formulation of radiotherapy regimens that could improve treatment outcomes. Whole-body PET/CT has high accuracy in detecting metastases. Chang et al. (23) reported a meta-analysis including 8 eligible studies. It illustrated the excellent performance of ^18^F-FDG PET/CT in the M staging of NPC. The pooled sensitivity, specificity, positive likelihood ratio, and negative likelihood ratio of ^18^F-FDG PET/CT were 0.83, 0.97, 23.38, and 0.19 respectively. The area under the curve was 0.9764 and the Q* index estimate was 0.9307. Results suggested that ^18^F-FDG PET/CT is reliable and effective in observing distant metastases in patients with NPC. However, the low negative likelihood ratio indicated that the negative result of ^18^F-FDG PET/CT is insufficient to exclude distant metastases, and other imaging examinations and clinical evidence are required for precise diagnosis.

Although ^18^F-FDG PET/CT has already been playing an important role in NPC staging, it still has certain limitations including poor detection of skull-base and intracranial metastases. Researchers tried to use novel radiotracers to improve the diagnostic ability of PET/CT. Previous studies have confirmed the feasibility and indicated that FAPI PET/CT provides advantages over ^18^F-FDG PET/CT in several diseases including head and neck cancers (24). The uptake of FAPI is higher in tumor lesions and lower in intracranial and other normal tissue areas than FDG due to their physiological distribution. Several studies explored the feasibility and potential advantage of FAPI PET/CT in patients with NPC. Zhao et al. (25) reported in their study that ^68^Ga-FAPI PET/CT showed higher tracer uptake than ^18^F-FDG PET/CT in primary tumors, regional lymph nodes, skeleton lesions, and visceral metastases for patients with NPC. 26% of patients upgraded TNM stage using ^68^Ga-FAPI PET/CT and 18% of patients changed radiotherapy plans. It confirmed the clinical utility of ^68^Ga-FAPI PET/CT for TNM staging and metastases detection in patients with NPC. Qin et al. (26) reported that no significant difference was observed between the uptake of FAPI in primary tumor lesions of NPC and that of FDG, whereas FAPI was superior to FDG in detecting skull-base and intracranial metastases in their study. The authors regarded that FDG reflected the glucose utilization of tumors and FAPI reflected the activity of the cancer-associated fibroblast. Therefore, FAPI could show an equivalent tumor detection capability to FDG and superiority for identifying skull-base and intracranial metastases. Summary of novel PET tracers in NPC management are displayed in **Table 2**.

**Table 2:** Critical studies investigating novel PET radiotracers in NPC.

Tracers	mechanism	Author (year)	Patient number	Clinical stage	Notes
FAPI	FAPI specifically binds to fibroblast activation protein which is highly expressed in the tumor microenvironment.	Zhao et al. (25) (2021)	45	II-III	^68^Ga-FAPI PET/CT showed higher radiotracer uptake than ^18^F-FDG PET/CT in primary tumors, regional lymph nodes, skeleton, and visceral metastases, and visualized larger tumor lesions for GTV. 26% of patients upgraded TNM stage and 18% of patients changed radiotherapy planning. ^18^F-FDG PET/CT visualized occult type of NPC which ^18^F-FDG PET/CT failed to detect.
		Qin et al. (26) (2020)	15	I-III	This study confirmed the feasibility of ^68^Ga-FAPI PET/MR in NPC. No significant difference was observed between FAPI and FDG uptake in primary tumor lesions. FAPI was superior to FDG in detecting skull-base and intracranial metastases.
NaF	NaF forms fluorapatite crystals to hydroxyapatite crystals in the extracellular bone matrix, which will be stimulated by bone remodeling.	Xiao et al. (27) (2020)	117	II-IV	^18^F-NaF PET/CT showed a satisfactory diagnostic value and performed excellently in detecting skull-base bone invasion and osseous metastases.
		Zhang et al. (28) (2018)	45	NA (pathologically confirmed)	^18^F-NaF PET/CT was superior to ^18^F-FDG PET/CT in detecting skull-base bone invasion and osseous metastases with better accuracy and sensitivity.
CHO	CHO is a precursor of phosphatidylcholine which component of cell membranes. Tumors with increased proliferation will stimulate its synthesis.	Jiang et al. (29) (2015)	14	NA	This study confirmed the feasibility of ^11^C-CHO PET/CT in radiotherapy planning. ^11^C-CHO fusion volumes were significantly larger than ^18^F-FDG volumes and derived a more consistent GTV delineation. ^11^C-CHO PET/CT could also detect intracranial and skull-base invasion.
FLT	FLT is a thymidine analog that is phosphorylated by thymidine kinase within cells and used for protein synthesis.	Hu et al. (30) (2020)	41	I-IV	This study confirmed the feasibility of ^18^F-FLT PET/CT in prognosis. Patients with MTV ≥8.6 and TLT ≥14.9 were significantly associated with lower LPFS, and the prognostic value of MTV and TLT was superior to SUVmax and MRI evaluation.

FAPI, fibroblast activation protein inhibitor; NaF, sodium fluoride; CHO, choline; FCH, fluorocholine; FLT, fluorothymidine; NPC, nasopharyngeal carcinoma; SUV, standardized uptake value; SUVmax, maximum standardized uptake value; GTV, gross tumor volume; MTV, metabolic tumor volume; TLT, total lesion thymidine; LPFS, local progression-free survival; NA, not applicable.

NaF is another novel radiotracer that has been used for identifying osseous metastases. ^99m^Tc-methylene diphosphonate (MDP) single photon emission computed tomography (SPECT) is routinely used as a noninvasive method for detecting osseous metastases in clinical practice but without cross-sectional images of all the lesions. ^18^F-NaF has similar biochemistry characteristics to ^99m^Tc-MDP. It can mainly replace the hydroxyl group in hydroxyapatite crystal and covalently binds bone surface. ^18^F-NaF has high sensitivity and bone affinity and shows better pharmacokinetic characteristics than ^99m^Tc-MDP including higher blood clearance rate and better bone tissue uptake resolution. Recent studies confirmed that ^18^F-NaF PET/CT has diagnostic value in detecting osseous metastases. It shows osteogenic and osteolytic metastases, micrometastases, and bone marrow metastases at the same time. ^18^F-NaF PET/CT could also observe skull-base involvement which ^18^F-FDG PET/CT is not able to detect. Those characteristics make ^18^F-NaF PET/CT equitable to be applied in patients with NPC. Xiao et al. (27) enrolled 117 patients with NPC staged II-IV. In their study, ^18^F-NaF PET/CT performed excellently in detecting skull-base bone invasion and osseous metastases. The sensitivity, specificity, accuracy, positive predictive value, and negative predictive value were 0.962, 0.947, 0.957, 0.974, 0.923 and 0.917, 0.957, 0.940, 0.936, 0.943 respectively. Another study conducted by Zhang et al. (28) also confirmed the feasibility of ^18^F-NaF PET/CT in the diagnosis of NPC and suggested the advantages in detecting skull-base and osseous metastases compared with ^18^F-FDG PET/CT. They demonstrated the accuracy and sensitivity for detecting skull-base and osseous metastases were significantly better than those of ^18^F-FDG PET/CT (92.9% *vs.* 51.9%; 98.3% *vs.* 42.9%). The superiority originates from the different imaging mechanisms between ^18^F-NaF and ^18^F-FDG. ^18^F-NaF PET/CT could reveal abnormal blood perfusion and bone reconstruction, and detect osteoblastic and osteolytic bone metastases with clear contrast revolution. Since skeleton is the most frequent metastatic site for NPC and occurs in 70–80% of patients with distant metastases, ^18^F-NaF PET/CT could provide significant value for diagnosis and staging. Future studies could focus on osseous metastases detection and compare the difference between ^18^F-NaF PET/CT, ^18^F-FDG PET/CT, and ^99m^Tc-MDP SPECT.

CHO PET/CT is especially utilized for the diagnosis of brain tumors and shows better visualization in many situations (31). Since ^18^F-FDG PET/CT cannot observe the intracranial and skull-base invasion in NPC, CHO PET/CT could make up for diagnosis. Jiang et al. (29) explored the feasibility and found that ^11^C-CHO PET/CT could help prevent the risk of NPC lesions that were not detected during diagnosis. They revealed that ^11^C-CHO PET/CT provided better information in detecting tumor invasion and could be an important complementary modality for ^18^F-FDG PET/CT in advanced NPC patients. Another study conducted by Wu et al. (32) also reported that ^11^C-CHO PET/CT showed better diagnostic accuracy for advanced NPC and higher sensitivity in detecting intracranial, skull base, and orbital invasion compared with ^18^F-FDG PET/CT. However, ^11^C-CHO has certain limitations including a short physical half-life and high uptake in many abdominal organs (32). Since the high uptake in the kidney and spleen has little effect on the diagnosis of NPC, the short physical half-life relatively restricts its use. ^18^F-CHO is an ^18^F-labeled choline analog synthesized with a longer half-life which is similar to ^18^F-FDG and considerable tracer uptake and has been used for malignancies including prostate cancer, breast carcinoma, and brain tumors. Several studies try to explore the potential advantages of ^18^F-CHO PET/CT in those conditions, but the feasibility in NPC patients still requires further investigations.

## PET/CT in the radiotherapy planning of NPC

Nasopharyngeal carcinoma is generally sensitive to radiotherapy which plays a significant role in the treatment of NPC. Radiotherapy alone (RT) is the main curative treatment for patients with early-stage NPC. Radiotherapy combined with chemotherapy is the main treatment for patients with locoregionally advanced NPC including concurrent chemoradiotherapy alone (CCRT) and concurrent chemoradiotherapy with adjuvant chemotherapy (ACT +CCRT) or induction chemotherapy (ICT+CCRT).

With the development of radiotherapy, new techniques including intensity-modulated radiotherapy (IMRT), intensity-modulated proton therapy (IMPT), and intensity-modulated carbon ion therapy (IMCT) continuously emerged. Whereas the more important element of successful radiotherapy planning is precise target delineation and exact dose delivery to tumor tissue. Gross tumor volume (GTV), clinical target volume (CTV), and planning target volume (PTV) are indicators commonly used for delineation. GTV represents the radiologically maximum tumor volume that is visible on imaging. CTV is the GTV plus margin of 5-10 mm that represents subclinical tumor and prophylactic lymph node region. PTV is the CTV plus margin of 3-4 mm that represents physical uncertainty during treatment. Precise delineation could direct radiation to damage tumor tissue effectively and reduce radiation injury simultaneously by the adjacent normal area. Supplemented by advanced radiotherapy and imaging techniques, radiotherapy has already become an effective weapon to cure NPC.

PET/CT has been widely applied in radiotherapy planning of various tumors as a functionally and metabolically based imaging modality and is also gradually used for patients with NPC. PET/CT guided radiotherapy could precisely display the edge of primary lesions of NPC and realize the accurate detection of the target. Huang et al. (33) reported that primary tumor volume measured by ^18^F-FDG PET/CT was significantly different from the volume measured by conventional CT in patients with NPC, indicating ^18^F-FDG PET/CT to be an effective imaging modality in determining the contours of the GTV for radiotherapy planning. Matsuura et al. (10) compared the clinical outcomes of patients with NPC undergoing IMRT guided by ^18^F-FDG PET/CT and CT. Results showed that ^18^F-FDG PET/CT accurately draw tumor delineation and achieved satisfactory clinical results. In addition, the application of ^18^F-FDG PET/CT significantly changed the target area up to 30-60% for radiotherapy. Although there was no significant difference in overall survival between the two groups, the recurrent rate in the radiation field guided by ^18^F-FDG PET/CT is significantly lower than in CT groups (11% *vs.* 31%, *p* < 0.01). Compared with CT, ^18^F-FDG PET/CT could improve the standardization of radiotherapy and reduce recurrence after IMRT as well as the risk of radiation injury. In Wu et al.’s (13) study, the impact of ^18^F-FDG PET/CT on the GTV for patients with early-stage NPC was similar to CT and MRI, whereas the impact on the PTV was significantly better. As the tumor delineation was magnified by ^18^F-FDG PET/CT on the PTV which was expanded from the GTV, it precisely avoided the underdosing. Since increasingly radiotherapy planning is computed according to the PTV, ^18^F-FDG PET/CT seems to be an indispensable modality for treatment planning. Nevertheless, Liu et al. (14) discussed the efficacy and toxicity of ^18^F-FDG PET/CT guided dose-painting IMRT in patients with locoregional advanced NPC. They reported that ^18^F-FDG PET/CT groups showed significantly better overall survival compared with CT groups (91.8% *vs.* 82.6%; *p* < 0.05) and no significant differences in toxicities were observed. They indicated that ^18^F-FDG PET/CT guided radiotherapy is an effective treatment modality with no increased toxicities for patients with locoregional advanced NPC. Parameters of PET/CT for target volume delineation in NPC are exhibited in **Table 3**.

**Table 3:** Different parameters of PET/CT for target volume delineation in NPC.

Target delineation strategies	Different threshold algorithm
Fixed absolute value method	SUV2.5 (threshold of SUV ≥2.5)
	SUV3.0 (threshold of SUV ≥3.0)
Fixed ratio range method	SUV40% (threshold of SUV ≥40% SUVmax)
	SUV50% (threshold of SUV ≥50% SUVmax)
	SUV60% (threshold of SUV ≥60% SUVmax)
(Semi-)Automated segmentation method	Adaptive relative threshold segmentation algorithm
	Gradient-based watershed segmentation algorithm
	Graph-based segmentation algorithm
	Halo-based contouring algorithm
	Anatomic biologic contouring algorithm

SUV, standardized uptake value; SUVmax, maximum standardized uptake value.

Novel PET radiotracers have also been used for radiotherapy planning including FAPI and CHO. As mentioned before, FAPI shows higher radiotracer uptake in primary tumors and lower uptake in normal tissue adjacent to tumor lesions than FDG (34). Therefore, the superior biodistribution pattern allows FAPI PET/CT to depict better GTV delineation than ^18^F-FDG PET/CT. Zhao et al. (25) reported that ^68^Ga-FAPI PET/CT visualized clearer tumor delineation than ^18^F-FDG PET/CT in both primary and metastatic lesions of NPC. The total tumor lesions measured by FAPI are significantly larger than total lesion glycolysis measured by FDG, and the uptake of FAPI in normal tissue is relatively lower. Hence ^18^F-FDG PET/CT provided additional information for GTV delineation and distinguish normal tissues more precisely, which could improve the efficacy of radiotherapy with lower toxicities. On the other side, the occult type of NPC which accounts for 30% of all patients is frequently difficult to visualize since it infiltrates and grows in the submucosa. ^18^F-FDG PET/CT is also unable to visualize and locate the primary tumor in more than half of the cases. In this study, ^68^Ga-FAPI PET/CT successfully detected the primary lesions that ^18^F-FDG PET/CT failed to locate in patients with the occult type of NPC. Results indicated that ^68^Ga-FAPI PET/CT could be used as an important complement to detect the occult type of NPC and improve radiotherapy outcomes.

The use of ^18^F-FDG PET/CT for GTV delineation occasionally underestimates tumor invasion in patients with locally advanced NPC. Oncologists are conservative about the uncertainty of tumor invasion including intracranial and skull-base invasion especially. CHO PET/CT could provide supplementary information for determining GTV of NPC. Jiang et al. (29) reported that ^11^C-CHO fusion volumes of NPC were significantly larger than ^18^F-FDG volumes and provided better radiotherapy targeting results in this study. ^11^C-CHO PET/CT prevented the risk of undetected tumor lesions of NPC during treatment. They also found this modality could detect intracranial and skull-base invasion which provided treatment planners with viable measurement for delineation. Meanwhile, ^11^C-CHO PET/CT reduced the inter-observer variation and obtained consistent GTV delineation compared. Therefore, the semi-automatic contouring results were better than those obtained by ^18^F-FDG PET/CT. This study suggested that ^11^C-CHO PET/CT could be more advantageous in GTV delineation of NPC than ^18^F-FDG PET/CT. However, the evidence in radiotherapy planning for patients with NPC is still insufficient. And more studies about PTV targeting results rather than GTV still need to be performed.

Precise tumor delineation is prominent for successful radiotherapy in NPC and current studies have confirmed that PET/CT could accurately detect tumor lesions and display the boundary. It is recognized that PET/CT is superior to plain CT and contrast-enhanced CT with better visualization of tumor, whereas MRI has better resolution in the nasopharyngeal region. Although PET/CT is more sensitive in the detection of NPC, the significant superiority for tumor delineation compared with MRI is still not proven. MRI remains a necessary imaging modality for radiotherapy planning. On the other hand, novel PET radiotracers including FAPI and CHO have been studied and showed significant advantages for GTV and PTV delineation. PET/CT provides additional metabolic information and makes it possible to deliver exact radiation to tumor tissue completely without misdiagnosis and radiation injury by normal tissue.

## PET/CT in the prognosis of NPC

Radiotherapy significantly improves the survival of patients with NPC, but there remains a significant rate of local failures and distant metastases after radiotherapy. Around 10% of patients have residual or recurrent disease after routine radiotherapy, especially those with locally advanced NPC. Early and accurate prognosis is essential for identifying patients who acquire more aggressive treatment options to improve radiotherapy outcomes. PET/CT could be used for prognosis prediction with the capability of reflecting metabolic information and biological characteristics of tumors.

Previous studies have revealed that patients with higher radiotracer uptake generally have inferior treatment outcomes and poor survival. Besides TNM stage, the standardized uptake value (SUV), metabolic tumor volume (MTV), and total lesion activity (TLA) are prognostic factors generally evaluated for NPC. Liu et al. (35) found patients having NPC with local or distant metastases had higher SUVmax than those without (7.4 *vs.* 4.5, *p* < 0.001). For those patients with metastases, SUVmax showed potential independent prognostic value related to treatment failure and survival. Xie et al. (36) also reported that SUVmax could be used as a prognostic factor for survival in patients with locally advanced NPC. Patients having NPC with SUVmax <8.0 before treatment had significantly better overall survival (*X^2^* = 5.53, *p* = 0.019) and disease-free survival (*X^2^* = 5.77, *p* = 0.016) than those with SUVmax ≥8.0. Nevertheless, Hung et al. (15) enrolled 371 patients with NPC of stage I-IV receiving different radiotherapy options and drew similar conclusions. In their study patients with SUVmax of primary tumor lesions ≥9.3 and SUVmax of lymph nodes ≥7.4 had significantly worse distant metastases free survival. They also found concurrent chemoradiotherapy with or without adjuvant chemotherapy could improve local and distant control. The results suggested that SUVmax of ^18^F-FDG PET/CT had effectively prognostic value and more aggressive systemic treatment could be considered in patients with high SUVmax.

MTV is defined as the sum of the metabolic volume with SUV above the cutoff values. TLA is defined as the product of the MTV and average SUV and is usually described as total lesion glycolysis (TLG) when FDG is used as the radiotracer. Fei et al. (16) reported that 3-year progression-free survival was significantly related to both SUVmax and MTV in patients with NPC. SUVmax ≥8.2 and MTV ≥4.0 were the cutoff values for worse survival and suggested more aggressive treatment. On the other side, Chan et al. (20) reported that patients having locally advanced NPC with the complete metabolic response of ^18^F-FDG PET/CT after radiotherapy showed better survival, and TLG could be an independent prognostic factor for overall survival with the cutoff value of TLG ≥330. Yoon et al. (17) enrolled 97 patients with NPC and demonstrated that TLG ≥322 could predict disease progression. High-dose radiotherapy could improve outcomes for those patients with high TLG.

Recently another prognostic factor that has been investigated is the heterogeneity of radiotracer uptake by tumors. Heterogeneity index (HI) is defined as the SUVmean divided by SUVmax. Ma et al. (18) enrolled 29 patients with local recurrence NPC (LRNPC) treated by carbon-ion radiotherapy (CIRT). Although conventional PET/CT parameters including SUVmax, MTV, and TLG showed limited prognostic values in this study, the HI was significantly effective for the prognosis of those patients. Patients with HI ≥0.81 had significantly worse local recurrence-free survival and progression-free survival. The authors considered that MTV and TLG were valuable predictors of tumor progression, and HI may be more related to radiotherapy response. MTV, TLG, and HI are parameters derived from SUVmax and could reflect tumor metabolic burden accurately and incorporate more information. Therefore, these parameters may be more optimal for the prediction of treatment outcomes compared with SUVmax.

FLT is a novel PET radiotracer used in NPC, which is correlated with cellular proliferation. Values of ^18^F-FLT PET/CT have been reported in prognosis in various malignancies including head and neck cancers (37), and studies for NPC have also emerged recently. Hu et al. (30) reported that ^18^F-FLT PET/CT could predict the prognosis of patients with locally recurrent NPC. In their study, MTV ≥8.6 and total lesion thymidine (TLT) ≥14.9 were significantly associated with lower local progression-free survival (adjusted HR: 5.59, *p* = 0.009; 7.76, *p* = 0.002). The prognostic value of MTV and TLT was superior to SUVmax and MRI evaluation. The results confirmed the feasibility of ^18^F-FLT PET/CT and suggested the potential superiority in prognosis. Since patients with locally recurrent NPC usually have worse outcomes, there is an urgent need to predict accurately the prognosis before and after radiotherapy. Qi et al. (38) also reported the prognostic value of ^18^F-FLT PET/CT in patients with NPC staged II-IV. However, ^18^F-FLT PET/CT showed no more visualized information compared with ^18^F-FDG PET/CT which is more strongly correlated to tumor regression. Thus ^18^F-FDG PET/CT is still the recommended modality for prognosis in the clinical process. ^18^F-FLT PET/CT is valuable for the prognosis of NPC but more studies concerning the feasibility and superiority need to be investigated.

PET/CT can effectively predict the prognosis of patients with NPC by its parameters including SUVmax, MTV, TLA, and HI. The essential of various PET-derived parameters is that the distribution of radiotracer uptake reflects tumor proliferation and invasion which influences treatment response and survival. Consequently, oncologists could determine radiotherapy options according to prognosis and ultimately improve the efficacy of radiotherapy. Moreover, novel radiotracers show huge potential value and could provide more information for prognosis. Further investigation could focus on this area and compare the efficacy between different radiotracers and parameters.

## PET/CT in the surveillance and assessment of NPC

The core of surveillance is to identify residual disease and recurrence after treatment. However, radiotherapy generally causes inflammatory fibrosis and apoptosis scar in the local nasopharyngeal region in patients with NPC. The post-radiation inflammatory response has similar imaging manifestations to tumor lesions, which makes it difficult to differentiate them by conventional imaging examination. With the capability of reflecting tumor metabolism, PET/CT is more accurate in distinguishing residual or recurrent disease from post-radiation changes (39). Liu et al. (40) reported a meta-analysis including 21 articles. They analyzed and compared the capability of ^18^F-FDG PET/CT in identifying residual disease and recurrence of NPC with that of CT and MRI. The results showed that the sensitivity, specificity, and diagnostic odds ratio for ^18^F-FDG PET/CT were all significantly higher than those of CT and MRI. The *Q* index for FDG PET/CT was significantly higher than those for CT and MRI (0.92, 0.72, 0.76, *p* < 0.01). This study confirmed the significant advantage of ^18^F-FDG PET/CT in disease surveillance after radiotherapy.

PET/CT has also been used for assessing radiotherapy response. It is a reliable post-treatment prognostic factor for patients with NPC. The significant decrease in tracer uptake is generally correlated with better survival. Jeong et al. (19) reported that ^18^F-FDG PET/CT within 6 months after completely radiotherapy had high negative-predictive values for predicting residual disease and recurrence in patients with NPC. ^18^F-FDG PET/CT played an important role in the assessment for treatment. In their study patients with SUVmax of lymph nodes ≥2.5 after radiotherapy had significantly higher risks of recurrence than those with lower SUVmax. Xie et al. (36) also reported that patients having NPC with metabolic complete response after radiotherapy had significantly better OS (*X^2^* = 5.12, *p* = 0.024) and DFS (*X^2^* = 5.54, *p* = 0.019) than patients with metabolic partial response. Nevertheless, Chan et al. (20) confirmed that ^18^F-FDG PET/CT was an independent prognostic indicator for patients with locally advanced NPC after CCRT and helped as guidance to individualize surveillance protocols. Those studies illustrated that ^18^F-FDG PET/CT could be used to evaluate the radiotherapy response of NPC and determine whether more aggressive salvage treatments are required.

There are few reports on other radiotracers besides FDG in disease surveillance and assessment of NPC. Some radiotracers including FAPI have been used for the detection of primary tumor lesions and metastases, but the ability to identify residual disease and recurrence is uncertain. Zhao et al. (25) investigated the detection abilities of ^68^Ga-FAPI PET/CT for post-radiotherapy patients with NPC. The results revealed that ^68^Ga-FAPI PET/CT showed a higher true-positive rate and lower true-negative rate in detecting local recurrence and metastases compared with ^18^F-FDG PET/CT. Two patients with false-positive uptake of FAPI were due to inflammatory fibrosis in the nasopharyngeal region after radiotherapy. In this respect, FDG is still the most appropriate radiotracer for identifying residual disease and recurrence. Moreover, the sample size of this study is small and further investigation of FAPI in disease surveillance is required.

Together with other conventional imaging methods, PET/CT is also unable to differentiate residual or recurrence disease from fibrosis tissue completely. Zhou et al. (41) reported a meta-analysis including 23 articles and analyzed the diagnostic ability of ^18^F-FDG PET/CT for residual or recurrence NPC. They found PET/CT has high sensitivity and specificity but significant heterogeneity in the diagnosis. An important explanation is that ^18^F-FDG uptake significantly increases due to inflammatory reaction after radiotherapy which creates false-positive findings. Therefore, clinical information and laboratory examinations are necessary to be considered for the diagnosis besides imaging findings. Meanwhile, novel radiotracers which could better identify tumors from fibrosis are urgently required to be investigated.

## Discussion

PET/CT has a growing role in radiotherapy for NPC. The feasibility and efficacy have been confirmed in the management of tumor diagnosis, radiotherapy planning, prognosis, surveillance, and assessment. PET/CT is accurate for TNM staging of NPC and identifying radiotherapy indications. In treatment planning, PET/CT could delineate target volume and exact dose delivery precisely. PET/CT also effectively predicts the prognosis of patients before and after radiotherapy and identifies residual disease and recurrence after treatment. With the progressive integration of anatomic and functional imaging, PET/CT propels the application of adaptive radiotherapy (ART) which delivers more personalized radiotherapy protocols in individual patients (3). PET/CT could visualize anatomic variations more precisely and adjust dose delivery to the target. Moreover, PET/CT could identify metabolic and functional variations in response to radiotherapy and conduct adaptive treatment modification. Compared with conventional examinations including CT and MRI, PET/CT shows irreplaceable value as a novel functionally and metabolically based imaging modality which is crucial guidance for adaptive radiotherapy in NPC.

^18^F-FDG PET/CT is routinely used for patients with NPC and is sensitive and effective in identifying tumor primary lesions and metastases. Therefore, it could be more accurate for tumor staging, target delineation, and residual tumor identification. On the other hand, coupled with metabolic information and different parameters, ^18^F-FDG PET/CT has unique diagnostic and prognostic value and directs more precise radiotherapy. However, ^18^F-FDG PET/CT also has certain limitations. Firstly, it is not able to observe skull-base and intracranial metastases which frequently occurs in NPC due to the physiological distribution of ^18^F-FDG. Secondly, its diagnostic ability for liver and skeleton metastases is not superior to MRI or SPECT. Finally, it is difficult to completely distinguish residual or recurrent tumors from post-radiation inflammation. Hence, novel PET radiotracers are urgently demanded to solve these clinical predicaments. FAPI, NaF, CHO, and FLT are radiotracers that have been used for NPC in recent research. They all show a lower distribution in brain tissue and have the potential abilities to observe skull-base and intracranial metastases. FAPI PET/CT has satisfactory detection capability and excellent imaging contrast between tumor and background. It could be used as an important complement, especially for tumor staging and radiotherapy planning. NaF PET/CT could be superior for identifying osseous metastases because NaF is highly associated with bone remodeling. CHO PET/CT and FLT PET/CT are ordinarily utilized for brain tumors and may show better visualization for primary lesions and metastases of NPC due to the tumor location.

Identifying post-radiation inflammation is vital for treatment assessment and projects following radiotherapy plans. The local inflammatory fibrosis and apoptosis scar caused by radiotherapy show similar imaging manifestations with residual tumor tissue and bring obstacles for oncologists to make a clinical decision. Current studies have confirmed that ^18^F-FDG PET/CT is superior to CT and MRI in distinguishing tumor and fibrosis with the ability to reveal tissue metabolism. However, the local inflammation also shows tracer uptake for a period after radiotherapy, which means assessment immediately after the completion of radiotherapy is extremely difficult (42). In general, ^18^F-FDG PET/CT is routinely performed 3 months after radiotherapy which is often recommended in clinical practice but unavoidably delays the treatment assessment. Therefore, novel radiotracers with lower uptake in post-radiation inflammation are valuable and may substitute ^18^F-FDG in the assessment after radiotherapy. Until now there are only a few reports focusing on post-radiation imaging by novel radiotracers including FAPI, but results showed the advantage was uncertain compared with ^18^F-FDG and the false-positive rate is high, which requires further investigation.

On the other aspect, contrast-enhanced PET/CT and PET/MR have increased in recent studies to eliminate those pitfalls. Contrast-enhanced PET/CT could have better imaging between contrast and background and reduce the missed diagnosis compared with conventional PET/CT which combines PET with plain CT in most cases. However, contrast-enhanced PET/CT has been rarely reported in patients with NPC. Similarly, PET/MRI aggregates the characteristics of both PET and MRI and provides metabolic imaging with excellent tissue resolution, especially for the nasopharyngeal region. In clinical practice, MRI has been more frequently used for radiotherapy due to its better tissue resolution. Therefore, PET/MRI seems more eligible for NPC and could detect tumor lesions more accurately which enables precise radiotherapy (43). A prospective study indicated that PET/MRI increased the accuracy of MRI and was more discernible than PET/CT in the staging of primary nasopharyngeal carcinoma (44). Results showed PET/MRI is superior to the combination of MRI and PET/CT. Moreover, another review summarized 25 clinical PET/MRI studies on head and neck cancer since 2015, reporting that PET/MRI was as least equally accurate as PET/CT and provided advantages over PET/CT in specific situations with lower radiation doses (45). However, PET/MRI has not been widely used in clinical practice, and research concerning target delineation for radiotherapy in NPC is still limited. PET/MRI is expected to serve as a better imaging modality and further investigation is required in the future.

The parameter derived from PET/CT is another important value for guiding radiotherapy in patients with NPC. Precise tumor target delineation is a key clinical concern and prominent for successful radiotherapy treatment. Current evidence shows that SUV is one of the most effective measurements for defining GTV, CTV, and PTV. In general, three segmentation strategies are used for target delineation: fixed absolute value method, the target volume is based on a range above a specific SUV threshold, such as SUV2.5 and SUV3.0; fixed ratio range method, the target volume is based on a range above a specific ratio of SUVmax, such as SUV40%, SUV50%, and SUV60%; (semi-)automated segmentation method, the target volume is based on computational analysis by different algorithms including adaptive relative threshold segmentation algorithm, gradient-based watershed segmentation algorithm, graph-based segmentation algorithm, halo-based contouring algorithm, and anatomic biologic contouring algorithm (32, 46–49). Several studies attempted to compare those methods for target delineation in patients with NPC. SUV2.5, SUV50%, and anatomic biologic contouring algorithm are relatively recommended for defining tumor volume. Moreover, the distribution of SUV makes it possible to carry out different radiation in one target area according to the biological characteristics of the tumor. It indicates that PET/CT is not only delineating tumor volume but describing cytological information inside the tumor, which enables it to delineate the biological target volume (BTV) and achieve more accurate individualized radiotherapy treatment. However, there is no consensus as to which delineation method unanimously proved to be standard. The definition of the threshold for NPC varies considerably among institutions and studies, partly because of the difference in imaging equipment, injection volume, and scanning time. Considering SUV reflects tumor biological information including metabolism, proliferation, cell perfusion, and density, it is accepted to be the most qualified parameter for delineation. Further investigation is required to standardize the image display protocol and threshold algorithm. On the other hand, parameters derived from PET/CT are also independent prognostic factors for NPC. Precise and early prognosis is important for treatment planning. SUVmax, MTV, TLA, and HI are parameters that have been confirmed, meanwhile, more potential parameters are being discovered by data analysis. Especially when we apply novel radiotracers to PET/CT, more information will be collected. For example, TLT derived from FLT PET/CT shows a better prognostic value than SUVmax (30). Radiomic features extracted from ^11^C-CHO were more robust than ^18^F-FDG with respect to segmentation and showed potential advantages for evaluating tumor heterogeneity and predicting treatment response (50). New parameters and quantification of them should be investigated to direct radiotherapy more precisely in patients with NPC.

Besides conventional radiotherapy which is based on γ-ray to damage tumor tissue, radionuclide (α, β, and auger electron based) could also be used as effective radiation therapy for patients with cancers, especially late-stage diseases with limited therapeutic alternatives (51, 52). PET/CT with different radiotracers is not only used for tumor imaging but also important guidance for targeted radionuclide therapy. For an instance, ^68^Ga-PSMA PET/CT and ^177^Lu-PSMA-617 have been used for imaging and therapy in patients with prostate cancer with increased prostate-specific membrane antigen expression (53). ^68^Ga-PSMA PET/CT provides precise imaging and distinguishes potential therapeutic patients for targeted β-particle therapy by ^177^Lu-PSMA-617. NPC is fundamentally sensitive to radiation, which provides a basis for radionuclide therapy. Furthermore, the utilization of the same radiotracer (*e.g.*, ^68^Ga-FAPI and ^90^Y-FAPI) in both PET/CT imaging and radionuclide therapy could combine diagnostic and therapeutic tools into a single platform, making it possible for personalized theranostic in patients with NPC. Although currently, no studies have found any radionuclide therapy effective for NPC, several radiotracers including FAPI have already been used for imaging and diagnosis. It is imaginable that those radiotracers could be facilitated to conduct targeted radionuclide therapy and effectuate theranostic in future investigation.

## Conclusion

PET/CT has been playing a crucial role in radiotherapy and is increasingly recognized as an irreplaceable imaging modality for diagnosis, target planning, prognosis, surveillance, and assessment of NPC. However, ^18^F-FDG PET/CT has limitations in observing skull-base and intracranial invasion, liver and skeleton metastases, and post-radiation inflammation. Novel PET radiotracers have been extensively investigated to compensate for those deficiencies but have insufficient evidence. More clinical trials about novel radiotracers with larger sample sizes as well as PET-derived parameters and specific quantification of tracer uptake should be conducted in future investigations.

## Author contributions

HL and ZK conceptualized this study and did the literature search. HL, YX, RZ and SL worked together to conduct the literature review and revise the manuscript draft. HL, ZK prepared the manuscript. XY, RZ and SL revised the manuscript. All authors contributed to the article and approved the submitted version.

## Funding

This study was supported by the Special Research Fund for Central Universities, Peking Union Medical College (Grant No. 3332022024).

## Conflict of interest

The authors declare that the research was conducted in the absence of any commercial or financial relationships that could be construed as a potential conflict of interest.

## Publisher’s note

All claims expressed in this article are solely those of the authors and do not necessarily represent those of their affiliated organizations, or those of the publisher, the editors and the reviewers. Any product that may be evaluated in this article, or claim that may be made by its manufacturer, is not guaranteed or endorsed by the publisher.
